# Association between castration-induced changes in circadian body temperature rhythms and gut microbiome diversity in goats

**DOI:** 10.1038/s41598-026-40455-0

**Published:** 2026-02-20

**Authors:** Ibuki Matsufuji, Yuri Kitagawa, Satoshi Ohkura, Yasuhiro Morita

**Affiliations:** 1 Department of Animal Sciences, Graduate School of Bioagricultural Sciences, Nagoya University, Nagoya, 464-8601 Japan; 2 Department of Integrative Physiology, Graduate School of Medicine, Nagoya University, Nagoya, 466-8550 Japan; 3 Department of Bioresource Sciences, Graduate School of Bioresource and Environmental Sciences, Kyushu University, Fukuoka, 819-0395 Japan

**Keywords:** Microbiology, Physiology, Zoology

## Abstract

Castration is a routine management practice in livestock production, used to control temperament, reproduction, and carcass traits. Beyond these intended outcomes, castration induces substantial physiological changes, particularly in energy metabolism and thermoregulation. Recent studies have revealed close interactions between host circadian physiology and the gut microbiome, suggesting that disturbances in this relationship may influence animal performance and welfare. However, the effects of castration-induced alterations in circadian body temperature (CBT) rhythms on intestinal microbial composition remain poorly understood in ruminants. This study investigated the association between castration, CBT rhythmicity, and gut microbiota composition in goats. Castration significantly modified CBT rhythms in both early- and late-castrated animals. Linear mixed-effects cosinor analysis demonstrated distinct changes in the amplitude and acrophase of CBT oscillations, with more pronounced effects observed in late-castrated goats. These physiological changes were accompanied by shifts in intestinal microbial β-diversity, as shown by distance-based redundancy analysis, suggesting that castration-induced alterations in host circadian regulation were associated with changes in microbial community structure. Given the small sample size, this study should be considered exploratory and hypothesis-generating. These findings provide new insights into the interplay between host circadian biology and the gut microbiome in ruminants. The present study may inform future research and management approaches aimed at improving animal health and productivity.

**Supplementary Information:**

The online version contains supplementary material available at 10.1038/s41598-026-40455-0.

## Introduction

Castration is a common management practice in livestock production, employed not only to control temperament and prevent unwanted reproduction but also to improve carcass quality, fat deposition, and meat palatability^1,2^. Beyond these production-oriented effects, castration induces substantial physiological changes, including alterations in energy metabolism and thermoregulation^1^. Such profound physiological changes have been demonstrated in rodent models, where orchidectomy reduces basal metabolic rate and core body temperature, effects that are reversible by testosterone replacement^3^, and in livestock, where castration modifies heat production, peripheral temperature distribution, and metabolic indicators^2,4^. In ruminants, these effects are further influenced by fermentative heat production in the rumen, which contributes substantially to thermal balance and may interact with endocrine status to modulate circadian body temperature rhythms^5,6^. Clarifying these thermal consequences in ruminants is therefore important, not only for improving animal welfare but also for optimizing production efficiency within the framework of eco-balanced livestock farming.

Body temperature exhibits robust circadian rhythms, reflecting the interplay of central and peripheral clocks, metabolic activity, and environmental cues^7^. These rhythms are critical for maintaining homeostasis and may serve as integrative signals linking host physiology to diverse biological systems. Disruption of circadian body temperature rhythms has been associated with metabolic dysregulation, altered energy balance, and reduced resilience to environmental stressors^8^.

Recent studies have highlighted a bidirectional relationship between host circadian physiology and the gut microbiome. The gut microbial community exhibits pronounced diurnal oscillations in both taxonomic composition and functional activity, largely entrained by host feeding–fasting cycles and circadian clock genes^9,10^. Disruption of circadian rhythms, for example through clock-gene mutations or altered light–dark cycles, induces microbial dysbiosis characterized by the loss of temporal structure and reduced microbial diversity^11,12^. Conversely, the microbiota plays an essential role in regulating host circadian physiology by producing metabolites such as short-chain fatty acids (SCFAs) and bile acid derivatives that act as zeitgebers for peripheral clocks and influence systemic energy metabolism^13^. These interactions have been linked to immune modulation, nutrient utilization, and the maintenance of metabolic homeostasis^14,15^. Thus, the circadian coordination between host and microbiota represents a fundamental axis of physiological regulation, and disruption of this synchrony can contribute to metabolic disease, inflammation, and reduced productivity in livestock species.

Disruptions in this host–microbiome relationship may therefore have downstream consequences for animal performance, disease resilience, and welfare, with indirect implications for the sustainability of livestock production systems. However, the extent to which castration-induced changes in circadian body temperature rhythms influence intestinal microbiota composition remains poorly understood in ruminants, and to our knowledge, no longitudinal study has directly examined these associations using continuous temperature monitoring combined with microbiome profiling.

The present study was exploratory and aimed to examine the association between castration-induced alterations in body temperature rhythms and gut microbiota composition in goats. By addressing this knowledge gap, we sought to clarify the physiological consequences of castration for thermoregulation and microbial ecology, and to provide insights into how interactions between heat production and the gut microbiota may relate to animal welfare and productivity, with potential indirect benefits for the sustainability of livestock production.

## Results

### Plasma testosterone

**Fig. 1:**
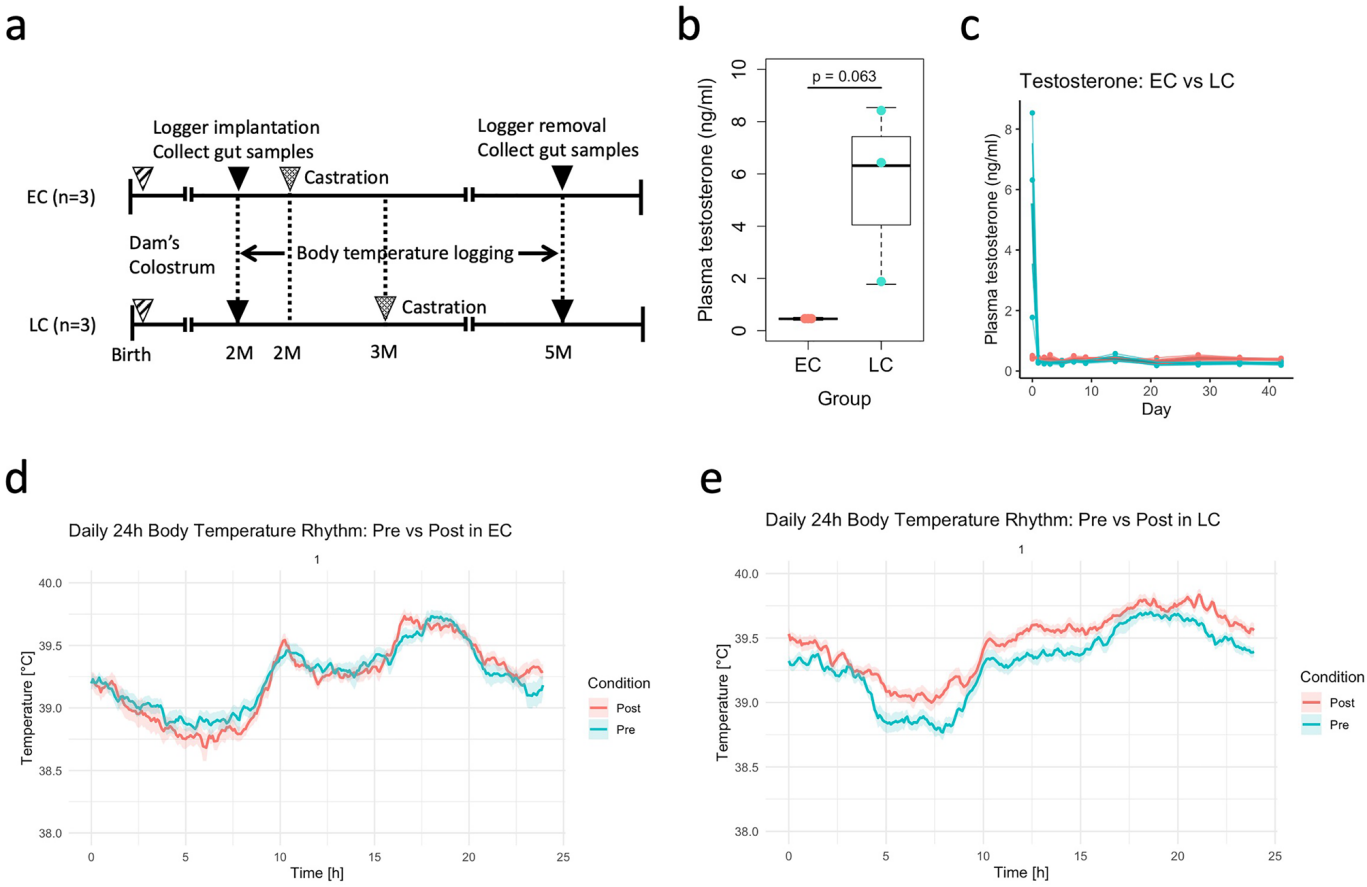
Experimental design, plasma testosterone concentrations, and body temperature rhythms in goats. (**a**) Schematic overview of the experimental design, showing grouping of goats based on castration timing and sample collection schedule. (**b**) Plasma testosterone concentrations before castration (d0) in early- (EC) and late-castrated (LC) goats. (**c**) Plasma testosterone concentrations after castration in EC and LC goats. (**d**) Body temperature rhythms in EC goats, analyzed using linear mixed-effects cosinor models. (**e**) Body temperature rhythms in LC goats, analyzed using linear mixed-effects cosinor models.

Plasma testosterone concentrations were analyzed using a linear mixed-effects model including Group and Day as fixed effects and Individual as a random effect. The model showed a significant main effect of Group (*p* = 0.044), whereas neither the effect of Day (*p* = 0.091) nor the Group × Day interaction (*p* = 0.103) reached statistical significance. On the day of castration (d0), plasma testosterone concentrations were 0.45 ± 0.04 ng/ml in EC and 6.32 ± 2.81 ng/ml in LC (mean ± SD). An exploratory unpaired t-test at d0 indicated a trend toward higher concentrations in LC (*p* = 0.063, Fig. 1b). On d1, concentrations were 0.43 ± 0.01 ng/ml in EC and 0.32 ± 0.03 ng/ml in LC. Thereafter, plasma testosterone levels remained below the detection limit in both groups (Fig. 1c).

### Changes in body temperature rhythms before and after castration

Body temperature rhythms were evaluated using linear mixed-effects cosinor models in both EC and LC goats (Fig. 1d,e). Body temperature rhythms differed significantly between pre- and post- castration periods in both EC and LC goats, although the magnitude of changes was greater in the LC group (Table 1). In the EC group, there was a significant effect of condition (pre- vs. post-castration) on mean body temperature (F = 12.17, *P* < 0.001). Strong 24 h rhythmicity was observed, with significant sinusoidal terms (sine and cosine components representing the daily oscillation; sin: F = 6385.94, *P* < 0.001; cos: F = 243.44, *P* < 0.001). Moreover, interactions between condition and rhythm terms were significant (Condition × sin: F = 66.79, *P* < 0.001; Condition × cos: F = 16.35, *P* < 0.001), indicating alterations in amplitude (half the peak-to-trough difference) and acrophase (timing of the daily peak) after castration. In the LC group, the effect of condition on mean body temperature was highly significant (F = 1152.74, *P* < 0.001). The circadian rhythm was again confirmed (sin: F = 10192.99, *P* < 0.001; cos: F = 309.53, *P* < 0.001), with significant interactions between condition and rhythm components (Condition × sin: F = 22.72, *P* < 0.001; Condition × cos: F = 8.93, *P* = 0.003).

**Table 1:** Summary of fixed-effect estimates (MESOR, amplitude, phase components) and type III ANOVA for body temperature rhythms in early-castrated (EC) and late-castrated (LC) goats, derived from linear mixed-effects cosinor models.

Group	Effect	Estimate	Std_Error	df	t_value	P_value	Type	F_value
EC	Intercept (MESOR)	39.22	0.044	2.02	888.03	< 0.001	Fixed effect	NA
	Condition (pre- vs. post-castration)	0.0206	0.0059	10,366	3.49	< 0.001	Fixed effect	NA
	sin24	− 0.367	0.0059	10,366	− 62.29	< 0.001	Fixed effect	NA
	cos24	− 0.048	0.0059	10,366	− 8.17	< 0.001	Fixed effect	NA
	Condition × sin24	0.068	0.0083	10,366	8.17	< 0.001	Fixed effect	NA
	Condition × cos24	− 0.034	0.0083	10,366	− 4.04	< 0.001	Fixed effect	NA
	Condition	NA	NA	NA	NA	< 0.001	ANOVA	12.17
	sin24	NA	NA	NA	NA	< 0.001	ANOVA	6385.94
	cos24	NA	NA	NA	NA	< 0.001	ANOVA	243.44
	Condition × sin24	NA	NA	NA	NA	< 0.001	ANOVA	66.79
	Condition × cos24	NA	NA	NA	NA	< 0.001	ANOVA	16.35
LC	Intercept (MESOR)	39.46	0.045	2.01	878.47	< 0.001	Fixed effect	NA
	Condition (pre- vs. post-castration)	− 0.157	0.0046	10,366	− 33.95	< 0.001	Fixed effect	NA
	sin24	− 0.315	0.0046	10,366	− 68.02	< 0.001	Fixed effect	NA
	cos24	0.048	0.0046	10,366	10.33	< 0.001	Fixed effect	NA
	Condition × sin24	− 0.031	0.0065	10,366	− 4.77	< 0.001	Fixed effect	NA
	Condition × cos24	0.02	0.0065	10,366	2.99	0.0028	Fixed effect	NA
	Condition	NA	NA	NA	NA	< 0.001	ANOVA	1152.74
	sin24	NA	NA	NA	NA	< 0.001	ANOVA	10,192.99
	cos24	NA	NA	NA	NA	< 0.001	ANOVA	309.53
	Condition × sin24	NA	NA	NA	NA	< 0.001	ANOVA	22.72
	Condition × cos24	NA	NA	NA	NA	0.0028	ANOVA	8.93

### Changes in gut microbiome composition before and after castration

**Fig. 2:**
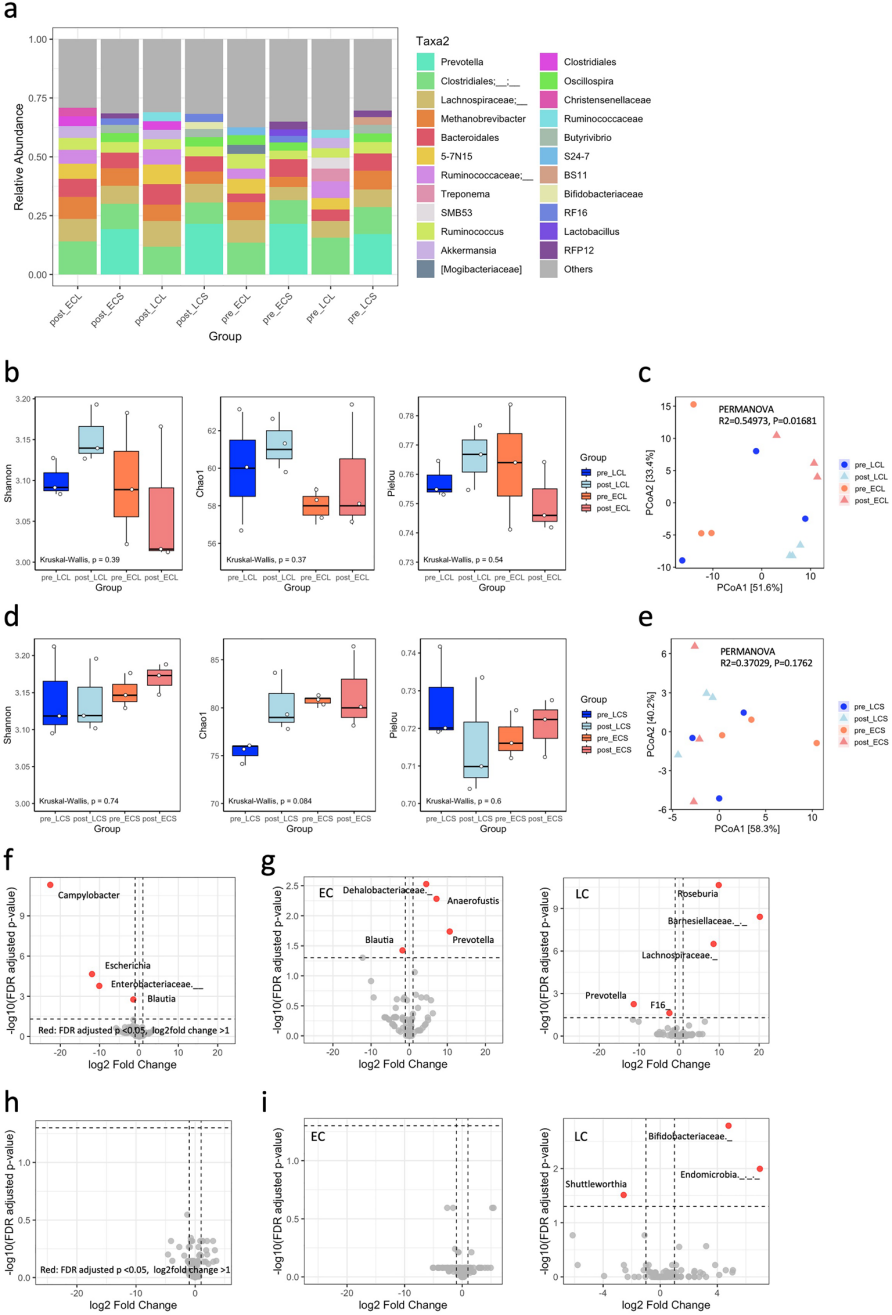
Gut microbiota composition in the colon and jejunum of goats. (**a**) Relative abundance of the top 10 most abundant genera in the colon (L) and jejunum (S) for early- (EC) and late-castrated (LC) goats. Remaining genera are grouped as “Others”. (**b**) Alpha diversity in the colon assessed by Shannon index, Chao1 richness, and Pielou’s evenness. (**c**) β-Diversity of colonic communities visualized using robust Aitchison distance and PCoA. (**d**) Alpha diversity in the jejunum assessed by Shannon index, Chao1 richness, and Pielou’s evenness. (**e**) β-Diversity of jejunal communities visualized using robust Aitchison distance and PCoA. (**f**–**i**), Differential abundance analysis using DESeq2 for the colon (**f**,**g**) and jejunum (**h**,**i**), shown separately for all animals and by group (EC vs. LC).

Relative abundance analysis of the intestinal microbiota at the genus level was performed separately for the colon and jejunum. The top 10 most abundant taxa were identified, with remaining genera collapsed into “Others” (Fig. 2a). Stacked bar plots revealed that the communities were dominated by a limited number of genera, including *Ruminococcus*, *Bacteroides*, *Prevotella*, and *Clostridium*, although their relative contributions varied between groups. In the colon, *Bacteroides* and *Ruminococcus* remained abundant across both EC and LC goats, while *Prevotella* tended to increase post-castration, particularly in the LC group. In the jejunum, *Clostridium* and *Lactobacillus* were dominant, and post-castration samples showed a tendency toward increased dominance of specific genera, suggesting reduced community evenness.

Alpha diversity in the colon, assessed by Shannon index, Chao1 richness, and Pielou’s evenness (Table S1), did not differ significantly between groups (Fig. 2b; Kruskal–Wallis, all *p* > 0.05; Dunn’s post-hoc tests, all pairwise comparisons ns). In contrast, β-diversity analysis using robust Aitchison distance revealed group-dependent differences (PERMANOVA: R^2^ = 0.55, *p* = 0.017; Fig. 2c, Table S2), with the largest dissimilarity observed between post-castration samples of EC and LC goats, although pairwise differences did not reach statistical significance after multiple testing correction (adjusted *p* = 0.15). In the small intestine, Shannon and Pielou’s evenness were comparable across groups. Chao1 richness showed *p* = 0.08 (Table S1), however, pairwise post-hoc comparisons were not significant (Fig. 2d). β-diversity analysis revealed no clear separation between groups (PERMANOVA: R^2^ = 0.37, *p* = 0.18; Fig. 2e, Table S2), indicating that overall community structures were largely maintained before and after castration.

Differential abundance analysis using DESeq2 identified taxa significantly altered by castration in the large intestine (Table S3). Across all animals, *Campylobacter* (log2FC = − 22.54, padj = 4.9 × 10^−12^), *Escherichia* (log2FC = − 11.93, padj = 2.2 × 10^−5^), and an unclassified *Enterobacteriaceae* (log2FC = − 10.08, padj = 1.7 × 10^−4^) were markedly reduced post-castration, while *Blautia* (log2FC = − 1.44, padj = 0.0017) also decreased (Fig. 2f). When analyzed by group (Fig. 2g), distinct patterns emerged: in LC goats, *Prevotella* (log2FC = − 11.36, padj = 0.0056) and TM7 (log2FC = − 2.43, padj = 0.024) were reduced, whereas *Roseburia* (log2FC = 9.89, padj = 2.2 × 10⁻¹¹), an unclassified *Lachnospiraceae* (log2FC = 8.61, padj = 3.1 × 10^−7^), and *Barnesiellaceae* (log2FC = 20.20, padj = 3.8 × 10^−9^) were enriched. In EC goats, *Prevotella* increased post-castration (log2FC = 10.63, padj = 0.018), while *Blautia* decreased (log2FC = − 1.75, padj = 0.038); additionally, *Dehalobacteriaceae* (log2FC = 4.46, padj = 0.0030) and *Anaerofustis* (log2FC = 7.15, padj = 0.0052) were enriched. In the small intestine, no significant taxa were detected when all animals were analyzed together (Fig. 2h). By group, only LC goats showed significant post-castration changes, including an unclassified *Bifidobacteriaceae* (log2FC = 4.78, padj = 0.0016), *Endomicrobia* (log2FC = 6.98, padj = 0.010), and *Shuttleworthia* (log2FC = − 2.56, padj = 0.031) (Fig. 2i, Table S3).

### Relationships between body temperature rhythms and gut microbiome diversity

**Fig. 3:**
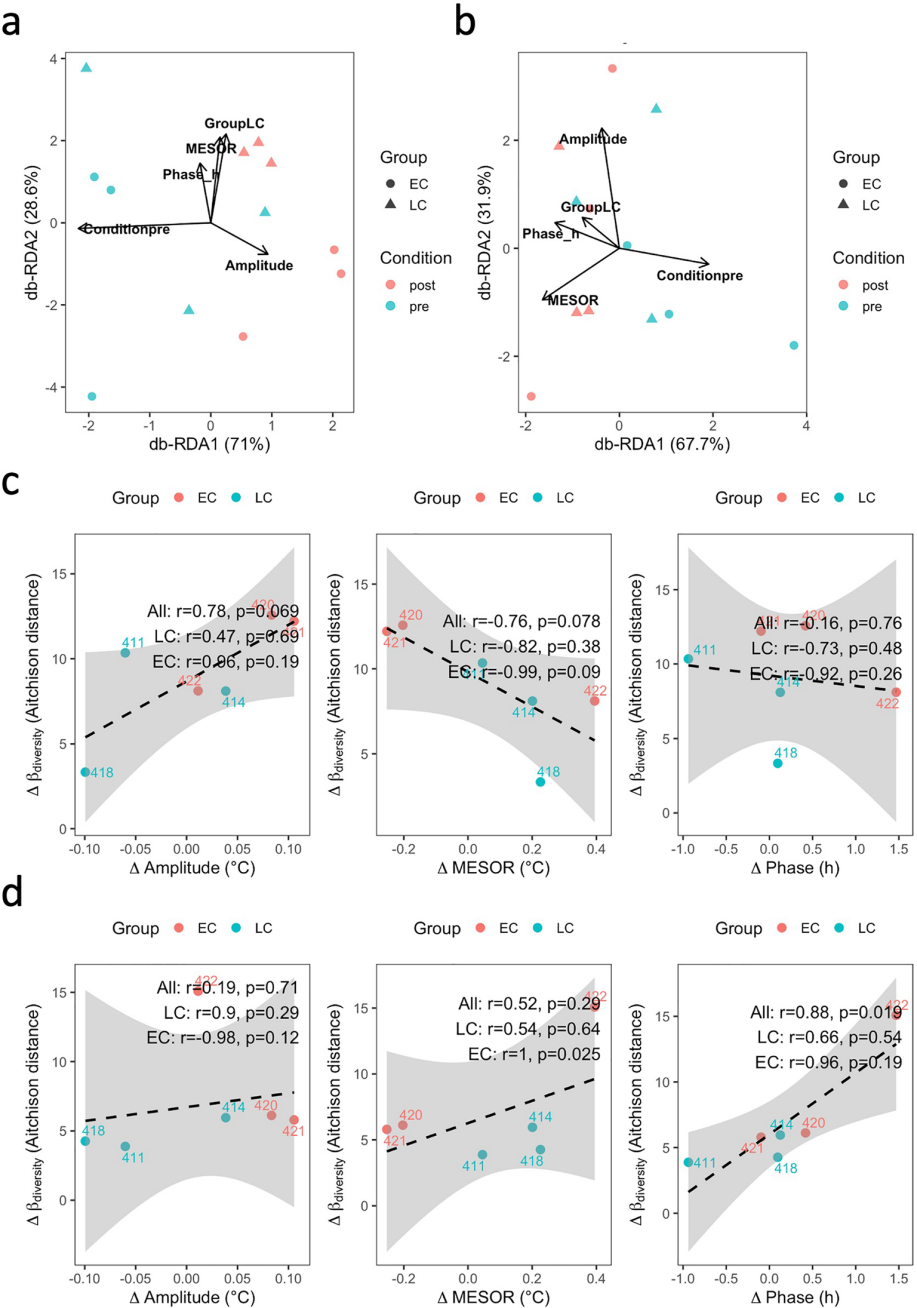
Relationships between circadian body temperature rhythms and gut microbial β-diversity in goats. (**a**) Distance-based redundancy analysis (db-RDA) of colonic microbial communities, illustrating the influence of pre/post castration condition, body temperature rhythm parameters (MESOR, amplitude, phase), and treatment group (EC vs. LC). (**b**) db-RDA of jejunal microbial communities, showing contributions of MESOR, amplitude, phase, condition, and group. (**c**) Correlations between pairwise pre/post differences (Δ) in body temperature rhythm parameters (MESOR, amplitude, phase) and Δβ-diversity in the colon. (**d**) Correlations between Δ body temperature rhythm parameters and Δβ-diversity in the jejunum.

In the large intestine (Fig. 3a, Table S4), distance-based redundancy analysis (db-RDA) indicated that pre/post condition significantly explained the variation in β-diversity (Aitchison distance) of the microbial community (capscale, F = 4.33, *p* = 0.034), whereas body temperature rhythm parameters (MESOR: midline estimating statistic of rhythm, representing the mean level of the fitted curve; amplitude: half the peak-to-trough difference; phase_h: acrophase expressed in hours on a 24-h scale) and treatment group (LC vs. EC) did not show significant effects (*p* > 0.18 for all). In the small intestine (Fig. 3b, Table S4), MESOR, amplitude, and phase yielded p-values of 0.068, 0.097, and > 0.14, respectively.

To assess whether changes in circadian body temperature rhythms corresponded to shifts in microbial community composition, we calculated the pairwise pre/post differences (Δ) in MESOR, amplitude, and phase_h for each animal and correlated these with the corresponding changes in β-diversity. In the large intestine (Fig. 3c), ΔMESOR and Δamplitude showed correlation coefficients of *r* = − 0.76 (*p* = 0.078) and *r* = 0.78 (*p* = 0.069), respectively, with Δβ-diversity, whereas Δphase was not correlated (*r* = − 0.16, *p* = 0.76). In contrast, in the small intestine (Fig. 3d, Δphase was positively correlated with Δβ-diversity (*r* = 0.88, *p* = 0.019), whereas ΔMESOR and Δamplitude were not associated (*p* > 0.28 for both).

## Discussion

Castration was associated with changes in circadian body temperature (CBT) rhythms in both early- and late-castrated goats. Linear mixed-effects cosinor analysis revealed distinct changes in the amplitude and acrophase of CBT oscillations, with more pronounced effects observed in late-castrated animals. These alterations were accompanied by shifts in intestinal microbial β-diversity, as shown by db-RDA, suggesting associations between castration-related physiological changes and differences in microbial community structures.

Castration was also associated with changes in circadian body temperature rhythms, particularly when performed after puberty, highlighting the role of gonadal hormones in stabilizing thermoregulatory oscillations in the present study. The link between testosterone withdrawal and altered rhythmicity suggests that endocrine signaling is linked to circadian regulation, and that the timing of castration influence the extent of disruption. Testosterone has been suggested to play a critical role in regulating CBT rhythms. Reduction of testosterone through castration has been reported to alter the amplitude and acrophase of CBT, particularly in post-pubertal animals, thereby disrupting circadian fluctuations in body temperature. Previous studies indicate that testosterone influences CBT rhythms, and that reductions in testosterone caused by castration lead to changes in CBT parameters, such as amplitude and acrophase^16,17^. Testosterone may influence CBT rhythms through central circadian regulators such as the suprachiasmatic nucleus (SCN) and peripheral thermogenic pathways, although the precise mechanisms remain unclear. Centrally, the SCN, the primary circadian pacemaker, expresses androgen and other gonadal steroid receptors, suggesting that testosterone may influence SCN-mediated rhythm regulation^18,19^. However, whether the SCN acts as a direct mediator of CBT changes remains unclear, and CBT can vary independently of other SCN-controlled rhythms^3,20^. These previous studies were conducted under controlled laboratory conditions and primarily focused on model animals. In contrast, the present study examined ruminant livestock across developmental stages and showed stage-dependent differences in circadian disruption, providing novel physiological evidence in livestock. On the other hand, peripherally, testosterone has also been implicated in adipose tissue thermogenesis, partly through regulation of uncoupling protein 1, which promotes non-shivering heat production^21^. Accordingly, the increases in body temperature and changes in amplitude observed after castration in the present study may partly reflect adipose tissue–derived thermogenesis independent of physical activity. Overall, testosterone appears to modulate CBT rhythms through multiple central and peripheral pathways, and its reduction may be associated with reduced stability of circadian body temperature.

Microbial responses in the cattle gut are region-specific, with the colon being more sensitive to host physiological changes than the small intestine, reflecting differential ecological and metabolic constraints across gut segments. The gut microbiome exhibits region-specific composition and diversity^22^, a phenomenon that can be explained by portfolio effects, where increased biodiversity within the ecosystem provides a functional buffer against environmental fluctuations^23^. Biogeography acts as a deterministic filter, selecting microbial populations through factors such as nutrient availability, chemical gradients, oxygen tension, and host secretions^24,25^Specifically, in the rumen, microbial taxa specialized in plant biomass degradation, including *Prevotella spp.* and *Fibrobacter spp*., are dominant, facilitating efficient hydrolysis^26,27^. In contrast, the small intestine shows a relative enrichment of spore-forming and aerobic bacteria, likely as an adaptation to environmental stresses such as oxygen exposure and host secretions^28,29^. In the colon, microbes adapted to the hindgut environment, including members of *Neocallimastigomycota* and *Alistipes*, predominate and perform carbon fixation and fermentation functions through metabolic pathways^22,30^. Consistent with these previous studies, the effects of castration-induced changes in body temperature were more evident in the colon, whereas no significant differences were detected in the small intestine. Moreover, microbiome profiles were derived from luminal contents rather than mucosal tissues, which may have limited sensitivity to detect region-specific host–microbe interactions, potentially underestimating true regional differences.

In the present study, no significant differences in α-diversity were detected, indicating that community composition shifted without an overall loss of diversity, a pattern consistent with ecological resilience and suggesting that the gut ecosystem maintained functional stability despite compositional restructuring. The resilience of the intestinal microbiota, defined as its ability to resist or recover from perturbations while maintaining core functions, has been reported in gut systems, where compositional changes occur without gross loss of diversity under environmental or physiological challenges^31^. In addition, studies in natural vertebrate populations have shown that β-diversity or composition shifts can be more closely linked to host outcomes than α-diversity alone, supporting the notion that maintenance of overall diversity can underlie functional stability despite compositional reorganization^32^. In mammals, both gut microbial community composition and functional metabolic profiles have been shown to be closely linked to host physiological traits^33^. Consistent with these studies, the microbiota changes observed in association with castration-related fluctuations in body temperature and hormones in the present study may reflect modulation of functional potential rather than impairment of ecosystem robustness.

SCFAs, particularly butyrate and acetate, serve as energy sources for intestinal epithelial cells and play crucial roles in maintaining mucosal barrier integrity and suppressing inflammation^34,35^. Moreover, SCFAs lower the luminal pH, thereby inhibiting the growth of pathogenic bacteria while promoting the colonization of commensal microbes^36^. Through these mechanisms, an increase in SCFA-producing bacteria may contribute to the intestinal environment toward a more stable state, supporting the maintenance of microbial homeostasis. Consistent with these　studies, the present study observed enrichment of SCFA-producing taxa together with reductions in opportunistic *Proteobacteria* after castration. Moreover, shifts in microbial composition in the present study favored enrichment of SCFA-producing taxa and reductions in opportunistic *Proteobacteria*, suggesting that castration may indirectly influence host–microbe metabolic interactions with potential benefits for gut health.

In the large intestine, the timing of castration appeared to critically influence microbial responses. LC goats exhibited a pronounced enrichment of SCFA–producing taxa such as *Roseburia*,* Lachnospiraceae*, and *Barnesiellaceae*, accompanied by marked reductions in *Proteobacteria* including *Escherichia* and *Campylobacter*, suggesting a shift toward a more metabolically favorable microbial profile. In contrast, EC goats showed more limited changes, including an increase in *Prevotella* and a decrease in *Blautia*, without the strong enrichment of SCFA producers observed in LC goats.

Previous studies have demonstrated that pubertal hormones, particularly testosterone, modulate gut microbial composition, and that reductions in androgen levels can promote reorganization of the gut microbiota toward SCFA-producing lineages^37,38^. SCFA-producing bacteria have also been implicated in the regulation of host thermogenesis and energy expenditure through gut–brain–adipose signaling pathways. Accordingly, the enrichment of SCFA producers observed in the present study may contribute to the stabilization of CBT rhythms via microbiota-derived metabolites. Moreover, the present findings extend these observations to ruminant livestock and further indicate that the developmental timing of castration modulates SCFA-related microbial responses within the gut microbiome. Collectively, these results suggest that the restructuring of colonic communities in response to castration may depend not only on the procedure itself but also on the developmental stage at which it occurs, thereby highlighting the importance of hormone–microbiome interactions in regulating gut ecosystem dynamics in ruminants. In addition, marked fluctuations in circulating testosterone levels during puberty may influence the gut microbiota.

Although the sample size was limited due to ethical and technical constraints and a non-castrated control group was not included, the study employed a within-individual longitudinal design, allowing each animal to serve as its own control and reducing inter-individual variability. Growth-related covariates (e.g., body weight or growth rate) were not incorporated into the statistical models because of the small sample size. Investigators were aware of group allocation because of the visible physiological status of the animals; however, all analyses were performed using coded datasets to minimize potential bias. Because this was a long-term continuous temperature monitoring study, ambient temperature effects could not be completely excluded and were not continuously recorded. All animals were housed under identical management conditions, thereby minimizing environmental variability. Developmental stage and surgical or handling stress may also have acted as confounders, and early- and late-castrated goats differed not only in hormonal exposure but also in maturation status, such that group differences may partly reflect developmentally contingent rather than purely hormone-driven responses. In addition, the small sample size may reduce statistical power and increase uncertainty in multivariate and differential abundance analyses, and these results should therefore be interpreted cautiously as hypothesis-generating rather than confirmatory. DESeq2-based differential abundance testing may be less robust with small sample sizes, potentially yielding unstable dispersion estimates, inflated log2 fold changes for low-abundance taxa, and an increased risk of false discoveries; therefore, functional inferences such as SCFA production should be considered putative without direct metabolite validation. Therefore, these findings should be interpreted as associative and exploratory, requiring confirmation in larger controlled studies.

While a clear association was identified between alterations in CBT rhythmicity and microbial community structure, causality cannot be inferred from the current data. Future interventions incorporating hormonal replacement or time-resolved metatranscriptomic analyses will be essential to determine whether temperature rhythms directly drive microbial restructuring or instead reflect underlying endocrine changes.

Our study demonstrates that castration-induced alterations in CBT rhythms are closely associated with region-specific shifts in the intestinal microbiota. In the colon, changes in the magnitude and mean level of CBT oscillations were associated with microbial community composition, whereas in the small intestine, the timing (phase) of CBT rhythms showed stronger associations with microbial alterations. These results highlight a coordinated interplay between host circadian physiology and intestinal microbiota, suggesting that temporal dynamics of CBT may play an important role in shaping microbial community structure.

Collectively, the present findings should be interpreted as associative and exploratory, provide new insights into relationships between host physiological rhythms and gut microbial ecosystems and a basis for future research on host–microbe interactions through the modulation of circadian biology. Moreover, monitoring circadian thermal rhythms may serve as a non-invasive biomarker of physiological adaptation and welfare in livestock, with potential applications in precision animal management under heat-stressed or metabolically challenged conditions. Because thermal rhythms were associated with shifts in gut microbial communities, continuous temperature monitoring may also act as an indirect indicator of internal microbiome dynamics, supporting health surveillance and precision livestock farming.

### Methods

The study was conducted from March to September, 2021 on a farm at the Field Science Center, Graduate School of Bioagricultural Sciences, Nagoya University (35° 06′ 42″ N, 137° 04′ 57″ E), Togo-town, Japan which maintains approximately 100 Shiba goats.

### Animal ethics

All experimental procedures were approved by the Committee of Care and Use of Experimental Animals of the Graduate School of Bioagricultural Sciences, Nagoya University (approval number: 2018031366). All animal experiments were performed in accordance with the relevant guidelines and regulations. The study is reported in accordance with the ARRIVE guidelines (https://arriveguidelines.org). No animals were euthanized during or after the experiment. All goats were returned to the experimental farm and maintained under standard husbandry conditions after the completion of the study. Sample size was determined based on ethical and logistical considerations rather than formal power analysis.

### Animals

Six male Shiba goats were used in this study (body weights in birth, 1.5–1.9 kg). All male Shiba goats were born and raised at the Field Science Center, Graduate School of Bioagricultural Sciences, Nagoya University, which served as the experimental site. All goats were born within the same breeding season and within a narrow time window (late February to early March 2021) and were therefore of similar age throughout the study period. The animals represented a single age-matched cohort, minimizing potential confounding effects of age-related physiological, circadian, and microbiome maturation. From birth until the end of the experiment, all goats were maintained in the same pen under identical husbandry conditions. Animals were fed a standardized diet consisting of dried timothy grass, oats, and hay cubes at prescribed amounts meet with nutrition requirement. Feeding was performed twice daily at approximately 09:00 and 17:00, and mineral supplements and water were available *ad libitum*. All animals remained healthy throughout the study.

### Experimental design

The experimental unit was an individual goat. Figure 1a provides a schematic overview of the experimental design, showing the grouping of goats based on castration timing and sample collection. At the beginning of the experiment, body weights of six Shiba goats were measured, and the animals were divided into two groups according to castration timing to observe subsequent physical changes. Based on the onset of puberty observed in this breed at approximately 11 kg body weight and three months of age, early-castrated (EC, *n* = 3) goats were castrated at around two months of age (body weight < 11 kg, range 5.5–8.7 kg). Late-castrated (LC, *n* = 3) goats were castrated at approximately three months of age (body weight ≥ 11 kg, range 11.3–13.3 kg). This grouping enabled comparison of pre- and post-pubertal castration to assess developmental stage–dependent physiological responses associated with pubertal hormonal changes. Animals were randomly assigned to EC or LC groups. Castration was performed by standard surgical orchiectomy under veterinary supervision. To evaluate circadian body temperature rhythms with high temporal resolution, abdominal temperature data loggers were implanted in a separate surgical procedure prior to castration, and core body temperature was continuously recorded until removal after castration. Continuous monitoring across multiple consecutive days was necessary to capture several complete circadian cycles and to obtain stable estimates of rhythm parameters such as amplitude and acrophase. Large and small intestinal contents were collected aseptically during the implantation (pre-castration) and de-implantation (post-castration) procedures of the temperature data loggers for subsequent microbiome analysis. Sampling at both time points enabled within-individual longitudinal comparisons, allowing each animal to serve as its own control and thereby reducing inter-individual variability.

### Implantation of abdominal thermos logger

Intra-abdominal temperature surrounding the intestines was continuously recorded every 5 min to an accuracy of better than 0.05 °C using data loggers (Bryn Logger; Bryn Owen Morgan Industries, Subiaco, Australia) implanted at 11 weeks of age. The loggers were calibrated against a certified precision platinum thermometer (certified by the Ando Keiki, Rhodes, Tokyo, Japan) between 33 and 42 °C and disinfected prior to surgery. Implantation was performed under ketamine–xylazine anesthesia (2.0 ml/10 kg BW). After a left flank incision, the logger was placed within the omentum (a fatty tissue covering the abdominal organs) and secured to the abdominal wall. The abdominal wall and skin were closed by routine suturing, and antibiotics (dihydrostreptomycin/benzylpenicillin suspension) were administered to prevent infection. At the end of the experiment, loggers were removed under anesthesia following the same procedure.

### Blood sampling for measuring plasma testosterone

Blood samples were collected from the jugular vein at 09:00 on days 0, 1, 2, 3, 5, 7, 14, 21, 28, 35, and 42 relative to castration. Plasma was separated by centrifugation (1500 g, 30 min, 4 °C) and stored at − 30 °C. Testosterone concentrations were measured using a commercial enzyme immunoassay kit (Testosterone EIA Kit; Funakoshi, Tokyo, Japan) according to the manufacturer’s instructions.

### 16 S rRNA gene sequencing of large and small intestinal samples

The microbiota of the large intestine (L, colon) and small intestine (S, jejunum) was analyzed by 16 S rRNA gene sequencing to characterize bacterial community composition. Sample processing, DNA extraction, library preparation, and taxonomic assignment based on a 16 S rRNA gene reference database, were conducted by Seibutsu Giken Co. (Sagamihara, Kanagawa, Japan) following the previous research (Suppl. text)^39^. After quality filtering and normalization, sequencing depth and total read counts were comparable across intestinal regions. All samples were processed using identical DNA extraction, library preparation, and sequencing protocols to minimize technical bias. Raw files of the bacterial V3–V4 16 S rRNA data have been deposited in the NCBI Sequence Read Archive (SRA) under BioProject accession number PRJNA1355188.

### Statistical analysis

No animals or data points were excluded from the analyses. The analyses were conducted using R software version 4.3.2 (http://www.R-project.org/), with the following packages; the results were deemed statistically significant at *p* ≤ 0.05. Prior to analysis, assumptions for parametric tests, including normality and homogeneity of variance, were evaluated. When these assumptions were not met, alternative approaches were applied, including linear mixed-effects models for repeated measures and non-parametric tests (Kruskal–Wallis with Dunn’s post-hoc tests). Microbiome compositional data were analyzed using distance- and permutation-based methods to minimize distributional assumptions.

### Plasma testosterone

To assess differences in plasma testosterone between EC and LC goats, we applied linear mixed-effects models (LMM) using the lmerTest package, with Group, Day, and their interaction as fixed effects and Individual as a random effect to account for repeated measurements. Significance was evaluated using type III ANOVA with Satterthwaite’s approximation for denominator degrees of freedom. Post-hoc comparisons were conducted with Tukey’s adjustment. In addition, unpaired t-tests were performed for day 0 comparisons.

### Body temperature analysis

Body temperature rhythms were analyzed separately for EC and LC goats using linear mixed-effects cosinor models incorporating 24 h sinusoidal terms (*sin* and *cos*). Although body temperature was recorded continuously from birth, only data from the 1-week period immediately before castration (pre-castration) and from 1 to 2 weeks after castration (post-castration) were included in the analysis. The first week post-castration was excluded as a recovery period. Period (pre- vs. post- castration) and its interactions with the sinusoidal terms were included as fixed effects, and animal ID was included as a random intercept to account for repeated measurements within individuals. This approach allowed estimation of MESOR, amplitude, and acrophase. Continuous temperature measurements recorded at 5-min intervals were analyzed within this mixed-effects framework to account for within-individual temporal dependence. Significance was evaluated by type III ANOVA with Satterthwaite’s approximation for denominator degrees of freedom.

### Microbiome analysis

All microbiome processing and statistical analyses were conducted using coded sample identifiers, and analysts were blinded to experimental group allocation to minimize potential bias. Alpha diversity metrics, including the Shannon index (diversity, “shannon”), Chao1 index (richness, “S.chao1”), and Pielou’s evenness index (evenness), were calculated to assess microbial diversity differences between EC and LC and pre- and post- castration samples. Group differences were evaluated using the Kruskal–Wallis (KW) test because of the non-normal distribution of these metrics. When a significant difference was detected, pairwise comparisons were performed using Dunn’s test with Benjamini–Hochberg (BH) correction to adjust for multiple testing. Because microbiome data are compositional, beta diversity was assessed using principal coordinate analysis (PCoA) based on robust Aitchison distances, calculated with the vegdist function using the “robust.aitchison” method, which is less sensitive to sparsity and zero inflation than conventional metrics. To evaluate the variation in microbiome composition attributable to sample grouping, permutational multivariate analysis of variance (PERMANOVA; adonis2) was performed with 100,000 permutations^39,40^. To further explore pairwise group differences, we performed a pairwise PERMANOVA with BH correction for multiple testing (pairwise adonis).

Differential abundance analysis of microbial taxa was conducted using DESeq2 (version 1.42.0) in R^41^. OTU count tables were filtered, normalized, and imported into DESeq2, with treatment group (EC vs. LC, pre- vs. post-castration) specified as the design factor. Wald tests were applied to evaluate pairwise contrasts between groups, including comparisons of pre- and post-castration within each treatment, as well as between EC and LC groups at corresponding time points. P-values were adjusted for multiple testing using the Benjamini–Hochberg false discovery rate (FDR), and taxa with adjusted p-values below 0.05 and absolute log2 fold change greater than 1 were regarded as significantly differentially abundant. Volcano plots were generated to visualize the distribution of log2 fold changes against adjusted p-values, with significant taxa highlighted.

### Integrated analysis with body temperature and microbiome before and after castration in both group

To evaluate the association between body temperature rhythms and gut microbiota composition, we performed distance-based redundancy analysis (db-RDA; vegan::capscale)^42^ using robust Aitchison distances as response variables and MESOR, amplitude, and phase (acrophase expressed in hours on a 24-h scale) as explanatory variables. Significance of individual terms was assessed by permutation tests. In addition, paired Δ-analysis was conducted. For each goat, the within-individual change in temperature rhythm parameters (ΔMESOR, ΔAmplitude, ΔPhase) between pre- and post-castration was calculated and correlated with the corresponding change in β-diversity (pre–post dissimilarity from the Aitchison distance matrix). Pearson correlation coefficients were computed separately for colonic and jejunal samples.

## Supplementary Information

Below is the link to the electronic supplementary material.

Supplementary Material 1

Supplementary Material 2

## Data Availability

The raw sequencing data have been deposited in the NCBI Sequence Read Archive (SRA) under BioProject accession number PRJNA1355188.
