# Supplementary material for: Association between castration-induced changes in circadian body temperature rhythms and gut microbiome diversity in goats
Source: Sci Rep. 2026 Feb 20;16:10058. doi: 10.1038/s41598-026-40455-0 (PMC13022122; doi:10.1038/s41598-026-40455-0)
Supplement: Supplementary file 2 — Supplementary Material 2 [file 41598_2026_40455_MOESM2_ESM.docx]

**Supplementary Methods**

***16S rRNA gene sequencing***

Collected samples were stored at -30 ºC, and sent to Seibutsu Giken Inc. (Kanagawa, Japan) for DNA extraction and NGS sequencing. Fecal samples were freeze-dried (VD-250R Freeze Dryer, Taitec, Kumagaya, Japan) and disrupted with glass beads by the Multi-beads Shocker (MB2200, Yasui-Kikai, Osaka, Japan) at 1500 rpm for 2min. The disrupted samples were lysed with lysis buffer (Lysis Solution F, Nippon Gene Co., Ltd. Tokyo, Japan) and allowed to stand at 65°C for 10 minutes. The samples were then centrifuged at 12,000 × g for 2 min, and the supernatant was separated for DNA extraction. DNA was purified from the aliquoted solution using Lab-Aid824s DNA Extraction kit (Zeesan Biotech, Xiamen, China).

The library was prepared from the purified samples using a 2-step tailed PCR method. The DNA was amplified by PCR using following primers according to Klindworth A., et al (2013). The bacterial 16S rRNA sequence of V3-V4 region was amplified using primers, first-step: V3-V4f_MIX (5ʹ-ACACTCTTTCCCTACACGACGCTCTTCCGATCT-NNNNN-CCTACGGGNGGCWGCAG-3ʹ) and V3-V4r_MIX (5ʹ-GTGACTGGAGTTCAGACGTGTGCTCTTCCGATCT-NNNNN-GACTACHVGGGTATCTAATCC-3ʹ), and second-step: 2ndF(5’-AATGATACGGCGACCACCGAGATCTACAC-Index2-ACACTCTTTCCCTACACGACGC-3’) and 2ndR (5’- CAAGCAGAAGACGGCATACGAGAT-Index1-GTGACTGGAGTTCAGACGTGTG-3’). PCR amplifications were carried out in a total volume of 20μL comprising, 1st: 5 μL of 2× PCR Buffer for KOD FX Neo (Toyobo, Osaka, Japan), 2 μL of dNTPs (each 2 mM), 0.2 μL of each primer (10 μM), 0.2 μL of KOD FX Neo (1.0 U/μL), and 1.0 μL of template DNA, 1.4  μL of Nuclease-Free Water; 2nd: 1.0 μL of 10x Ex Buffer for ExTaq HS (Takara Bio, Otsu, Japan), 0.8 μL of dNTPs (each 2.5 mM), 0.5 μL of each primer (10 μM), 2.0 μL of PCR product, 0.1 μL of ExTaq HS (5 U/μL), and 5.1 μL of Nuclease-Free Water. The PCR conditions were as follows, 1st: initial denaturation at 94°C for 2 min, followed by 30 cycles of denaturation at 98°C for 10 s, annealing at 55°C for 30 s, extension at 68°C for 30 s, and final extension at 68°C for 7 min; 2nd: initial denaturation at 94°C for 2 min, followed by 10 cycles of denaturation at 94°C for 30 s, annealing at 60°C for 30 s, extension at 72°C for 30 s, and final extension at 72°C for 5 min.

The concentration of the prepared library was measured using a microplate fluorescence reader (Synergy H1, Bio Tek, Winooski, VT, USA) and QuantiFluor dsDNA System (Promega, Madison, WI, USA) following the manufacturer’s instructions. The quality of the prepared libraries was examined using Fragment Analyzer and dsDNA 915 Reagent Kit (Agilent Technologies). Sequencing was performed using the MiSeq system with MiSeq Reagent Kit v3 (Illumina, San Diego, CA, USA) at 2×300 bp. Using Fastq_barcode_splitter of Fastx toolkit (ver. 0.0.14), only sequences that matched exactly with the primer were extracted and sequences with low quality score (< 20) were removed leaving a final base pair length of 130 or less. Pre- processed sequences were then analyzed in Quantitative Insights into Microbiology Ecology (QIIME) 2 (v2022.8) and clustered into Operational Taxonomic Units (OTUs) using Greengenes database (https://greengenes.lbl.gov/Download/) at 97% similarity.
